# Supplementary material for: Binding stoichiometry and structural model of the HIV-1 Rev/importin β complex
Source: Life Sci Alliance. 2022 Aug 22;5(10):e202201431. doi: 10.26508/lsa.202201431 (PMC9396022; doi:10.26508/lsa.202201431)
Supplement: Supplementary file 1 [file LSA-2022-01431_TableS1.docx]

**Table S1. Quality control of Impβ and Rev constructs by LC/ESI mass spectrometric analysis.**

| **Protein** | **Mutations** | **Expected**  **average mass**  **(Da)** | **Observed**  **average mass**  **(Da)** | Δ **mass**  **(Observed - Expected)**  **(Da)** |  |
| --- | --- | --- | --- | --- | --- |
| Impβ WT | - | 97,298.6 | 97,299.6 | 1.0 |  |
| **His-Impβ:** |  |  |  |  |  |
| Impβ WT | - | 100,296.9 | 100,299.2 | 2.3 |  |
| ImpB B1 | E152R E203R E274R E281R | 100,405.2 | 100,403.3 | -1.9 |  |
| ImpB B2 | D288R E289R D292R E299R | 100,433.3 | 100,432.4 | -0.9 |  |
| ImpB B3 | D339R D340R | 100,379.1 | 100,373.4 | -5.7 |  |
| ImpB B4 | E437R E479R E534R | 100,378.1 | 100,380.3 | 2.2 |  |
| ImpB B5 | E483R D486R D490R | 100,405.9 | 100,404.2 | -1.7 |  |
| ImpB B6 | E530R D579R E626R | 100,390.1 | 100,392.2 | 2.1 |  |
| ImpB B7 | D753R D756R E760R | 100,405.9 | 100,404.2 | -1.7 |  |
| ImpB D288R | D288R | 100,338.0 | 100,342.6 | 4.6 |  |
| ImpB E289R | E289R | 100,324.0 | 100,328.3 | 4.3 |  |
| ImpB D292R | D292R | 100,338.0 | 100,343.0 | 5.0 |  |
| ImpB E299R | E299R | 100,324.0 | 100,327.5 | 3.5 |  |
| ImpB D339R | D339R | 100,338.0 | 100,340.2 | 2.2 |  |
| ImpB D340R | D340R | 100,338.0 | 100,340.9 | 2.9 |  |
| ImpB E437R | E437R | 100,324.0 | 100,331.7 | 7.7 |  |
| ImpB E479R | E479R | 100,324.0 | 100,331.2 | 7.2 |  |
| ImpB E534R | E534R | 100,324.0 | 100,331.7 | 7.7 |  |
| **Rev:** |  |  |  |  |  |
| Rev WT | - | 13,175.9 | 13,176.9 | 1.0 |  |
| Rev OD* | V16D 155N | 13,263.8 | 13,263.9 | 0.1 |  |
| Rev ODΔ | V16D 155N | 8,030.9 | 8,031.0 | 0.1 |  |
| Rev R1 | R14D R17D K20D | 13,080.6 | 13,080.4 | -0.2 |  |
| Rev R2 | R35D R38D R39D | 13,052.6 | 13,050.6 | -2.0 |  |
| Rev R3 | R41D R44D R48D | 13,052.6 | 13,052.4 | -0.2 |  |
| Rev R4 | R42D R43D R46D | 13,052.6 | 13,052.1 | -0.5 |  |
| Rev R5 | R50D R58D | 13,093.7 | 13,093.3 | -0.4 |  |
| Rev R35D | R35D | 13,134.8 | 13,134.9 | 0.1 |  |
| Rev R38D | R38D | 13,134.8 | 13,134.1 | -0.7 |  |
| Rev R39D | R39D | 13,134.8 | 13,134.0 | -0.8 |  |
| Rev R41D | R41D | 13,134.8 | 13,135.0 | 0.2 |  |
| Rev R42D | R42D | 13,134.8 | 13,135.1 | 0.3 |  |
| Rev R43D | R43D | 13,134.8 | 13,134.8 | 0.0 |  |
| Rev R44D | R44D | 13,134.8 | 13,135.1 | 0.3 |  |
| Rev R46D | R46D | 13,134.8 | 13,134.8 | 0.0 |  |
| Rev R48D | R48D | 13,134.8 | 13,135.1 | 0.3 |  |
| * The Rev^OD^ construct contains an additional Ala residue after the TEV cleavage site. | | | | | |
