# Supplementary material for: Binding stoichiometry and structural model of the HIV-1 Rev/importin β complex
Source: Life Sci Alliance. 2022 Aug 22;5(10):e202201431. doi: 10.26508/lsa.202201431 (PMC9396022; doi:10.26508/lsa.202201431)
Supplement: Supplementary file 2 [file LSA-2022-01431_TableS2.docx]

**Table S2. Summary of IC_50_ values determined in FP competition assays^1^.**

| **Rev** | **WT** | | | **R1** | | | **R2** | | | **R3** | | | **R4** | | | **R5** | |
| --- | --- | --- | --- | --- | --- | --- | --- | --- | --- | --- | --- | --- | --- | --- | --- | --- | --- |
| IC50 (μM) | Mean ± SD | N | Mean ± SD | | N | Mean ± SD | | N | Mean ± SD | | N | Mean ± SD | | N | Mean ± SD | | N |
| **Impβ WT** | 0.502 ± 0.022 | 34 | 1.609 ± 0.052 | | 6 | 4.971 ± 0.268 | | 16 | 4.860 ± 0.325 | | 22 | 4.852 ± 0.134 | | 13 | 0.846 ± 0.023 | | 6 |
| **ImpB B1** | 0.517 ± 0.041 | 6 | 1.399 ± 0.091 | | 4 | 2.994 ± 0.250 | | 6 | 2.873 ± 0.130 | | 6 | 2.840 ± 0.126 | | 6 | 0.549 ± 0.055 | | 4 |
| **ImpB B2** | 0.549 ± 0.086 | 12 | 1.414 ± 0.061 | | 4 | 1.649 ± 0.157 | | 12 | 1.703 ± 0.208 | | 11 | 1.533 ± 0.117 | | 14 | 0.844 ± 0.095 | | 4 |
| **ImpB B3** | 0.431 ± 0.050 | 12 | 1.481 ± 0.048 | | 4 | 4.185 ± 0.496 | | 10 | 3.298 ± 0.562 | | 12 | 3.226 ± 0.213 | | 6 | 0.670 ± 0.035 | | 4 |
| **ImpB B4** | 0.441 ± 0.076 | 14 | 1.385 ± 0.022 | | 4 | 4.188 ± 0.493 | | 6 | 3.145 ± 0.446 | | 14 | 3.080 ± 0.260 | | 6 | 0.665 ± 0.011 | | 4 |
| **ImpB B5** | 0.440 ± 0.021 | 6 | 1.295 ± 0.064 | | 4 | 4.854 ± 0.509 | | 6 | 4.807 ± 0.541 | | 5 | 4.444 ± 0.304 | | 6 | 0.844 ± 0.056 | | 4 |
| **ImpB B6** | 0.433 ± 0.065 | 6 | 1.584 ± 0.174 | | 4 | 4.538 ± 0.720 | | 6 | 3.865 ± 0.430 | | 6 | 3.960 ± 0.494 | | 6 | 0.643 ± 0.070 | | 4 |
| **ImpB B7** | 0.440 ± 0.082 | 6 | 1.575 ± 0.171 | | 4 | 4.849 ± 0.849 | | 6 | 4.727 ± 0.749 | | 6 | 4.404 ± 0.655 | | 6 | 0.866 ± 0.025 | | 4 |

| **Rev** | **WT** | | | **R2** | | | **R35D** | | | **R38D** | | | **R39D** | | |
| --- | --- | --- | --- | --- | --- | --- | --- | --- | --- | --- | --- | --- | --- | --- | --- |
| IC50 (μM) | Mean ± SD | N | Mean ± SD | | N | Mean ± SD | | N | Mean ± SD | | N | Mean ± SD | | N |  |
| **Impβ WT** | 0.502 ± 0.022 | 34 | 4.971 ± 0.268 | | 16 | 0.883 ± 0.028 | | 10 | 0.802 ± 0.023 | | 10 | 0.928 ± 0.048 | | 10 |  |
| **ImpB B2** | 0.549 ± 0.086 | 12 | 1.649 ± 0.157 | | 12 | 0.788 ± 0.146 | | 6 | 0.771 ± 0.092 | | 6 | 0.918 ± 0.107 | | 6 |  |
| **ImpB D288R** | 0.629 ± 0.031 | 6 | 2.482 ± 0.161 | | 6 | 0.926 ± 0.149 | | 6 | 0.832 ± 0.125 | | 5 | 0.998 ± 0.059 | | 6 |  |
| **ImpB E289R** | 0.530 ± 0.070 | 6 | 4.139 ± 0.184 | | 6 | 0.820 ± 0.076 | | 6 | 0.744 ± 0.073 | | 6 | 0.855 ± 0.074 | | 6 |  |
| **ImpB D292R** | 0.531 ± 0.057 | 6 | 3.658 ± 0.109 | | 6 | 0.752 ± 0.027 | | 6 | 0.664 ± 0.036 | | 6 | 0.794 ± 0.042 | | 6 |  |
| **ImpB E299R** | 0.495 ± 0.067 | 6 | 4.487 ± 0.562 | | 6 | 0.738 ± 0.029 | | 6 | 0.722 ± 0.088 | | 6 | 0.810 ± 0.059 | | 6 |  |

| **Rev** | **WT** | | | **R3** | | | **R41D** | | | **R44D** | | | **R48D** | |
| --- | --- | --- | --- | --- | --- | --- | --- | --- | --- | --- | --- | --- | --- | --- |
| IC50 (μM) | Mean ± SD | N | Mean ± SD | | N | Mean ± SD | | N | Mean ± SD | | N | Mean ± SD | | N |
| **Impβ WT** | 0.502 ± 0.022 | 34 | 4.860 ± 0.325 | | 22 | 0.828 ± 0.017 | | 15 | 0.607 ± 0.021 | | 14 | 0.551 ± 0.030 | | 15 |
| **ImpB B2** | 0.549 ± 0.086 | 12 | 1.703 ± 0.208 | | 11 | 0.604 ± 0.112 | | 8 | 0.592 ± 0.037 | | 4 | 0.541 ± 0.124 | | 4 |
| **ImpB D288R** | 0.629 ± 0.031 | 6 | 2.398 ± 0.383 | | 4 | 0.741 ± 0.189 | | 8 | 0.740 ± 0.053 | | 4 | 0.547 ± 0.125 | | 4 |
| **ImpB E289R** | 0.530 ± 0.070 | 6 | 4.046 ± 0.979 | | 4 | 0.678 ± 0.181 | | 8 | 0.671 ± 0.066 | | 4 | 0.495 ± 0.147 | | 4 |
| **ImpB D292R** | 0.531 ± 0.057 | 6 | 3.804 ± 1.054 | | 4 | 0.687 ± 0.205 | | 8 | 0.688 ± 0.110 | | 4 | 0.501 ± 0.157 | | 4 |
| **ImpB E299R** | 0.495 ± 0.067 | 6 | 5.428 ± 1.153 | | 4 | 0.831 ± 0.166 | | 8 | 0.692 ± 0.064 | | 4 | 0.495 ± 0.062 | | 4 |
| **ImpB B3** | 0.431 ± 0.050 | 12 | 3.298 ± 0.562 | | 12 | 0.691 ± 0.173 | | 8 | 0.495 ± 0.074 | | 6 | 0.329 ± 0.083 | | 8 |
| **ImpB D339R** | 0.435 ± 0.095 | 6 | 3.125 ± 0.702 | | 6 | 0.694 ± 0.146 | | 8 | 0.554 ± 0.039 | | 6 | 0.491 ± 0.081 | | 8 |
| **ImpB D340R** | 0.537± 0.126 | 6 | 4.702 ± 0.982 | | 6 | 0.860 ± 0.223 | | 8 | 0.689 ± 0.133 | | 6 | 0.550 ± 0.102 | | 8 |
| **ImpB B4** | 0.441 ± 0.076 | 14 | 3.145 ± 0.446 | | 14 | 0.700 ± 0.134 | | 10 | 0.490 ± 0.116 | | 8 | 0.316 ± 0.092 | | 8 |
| **ImpB E437R** | 0.394 ± 0.079 | 8 | 3.753 ± 0.397 | | 8 | 0.672 ± 0.166 | | 10 | 0.493 ± 0.134 | | 8 | 0.407 ± 0.122 | | 8 |
| **ImpB E479R** | 0.509 ± 0.092 | 4 | 4.510 ± 1.587 | | 8 | 0.888 ± 0.107 | | 6 | 0.763 ± 0.092 | | 4 | 0.585 ± 0.032 | | 4 |
| **ImpB E534R** | 0.350 ± 0.018 | 5 | 4.311 ± 0.643 | | 7 | 0.711 ± 0.147 | | 9 | 0.581 ± 0.116 | | 7 | 0.481 ± 0.127 | | 8 |

| **Rev** | **WT** | | | **R4** | | | **R42D** | | | **R43D** | | | **R46D** | |
| --- | --- | --- | --- | --- | --- | --- | --- | --- | --- | --- | --- | --- | --- | --- |
| IC50 (μM) | Mean ± SD | N | Mean ± SD | | N | Mean ± SD | | N | Mean ± SD | | N | Mean ± SD | | N |
| **Impβ WT** | 0.502 ± 0.022 | 34 | 4.852 ± 0.134 | | 13 | 0.646 ± 0.079 | | 8 | 0.583 ± 0.027 | | 8 | 0.579 ± 0.011 | | 8 |
| **ImpB B2** | 0.549 ± 0.086 | 12 | 1.533 ± 0.117 | | 14 | 0.481 ± 0.120 | | 8 | 0.489 ± 0.052 | | 8 | 0.440 ± 0.070 | | 8 |
| **ImpB D288R** | 0.629 ± 0.031 | 6 | 2.065 ± 0.210 | | 8 | 0.254 ± 0.026 | | 6 | 0.387 ± 0.134 | | 8 | 0.272 ± 0.023 | | 6 |
| **ImpB E289R** | 0.530 ± 0.070 | 6 | 3.975 ± 0.169 | | 8 | 0.539 ± 0.073 | | 8 | 0.522 ± 0.047 | | 8 | 0.452 ± 0.028 | | 7 |
| **ImpB D292R** | 0.531 ± 0.057 | 6 | 3.907 ± 0.876 | | 8 | 0.580 ± 0.143 | | 8 | 0.515 ± 0.081 | | 8 | 0.476 ± 0.105 | | 8 |
| **ImpB E299R** | 0.495 ± 0.067 | 6 | 4.090 ± 0.218 | | 7 | 0.494 ± 0.152 | | 8 | 0.440 ± 0.120 | | 8 | 0.417 ± 0.077 | | 8 |

**^1^** Experimental values shown more than once are in black the first time they appear in the table and in grey upon subsequent instances.
