# Supplementary material for: Binding stoichiometry and structural model of the HIV-1 Rev/importin β complex
Source: Life Sci Alliance. 2022 Aug 22;5(10):e202201431. doi: 10.26508/lsa.202201431 (PMC9396022; doi:10.26508/lsa.202201431)
Supplement: Supplementary file 3 [file LSA-2022-01431_TableS3.docx]

**Table S3. Summary of initial HADDOCK docking experiments with Rev at the N- or C-site.**

**A. Docking parameters**

| **General parameters** |  |
| --- | --- |
| PDB entries used | 2X7L (Rev), 1UKL (Impβ) |
| Active residues^(1)^ | R42^Rev^, R46^Rev^, D288^Impβ^ with (experiments 3-4) or without (experiments 1-2) E437^Impβ^ |
| Passive residues^(1)^ | All solvent-accessible residues (except those designated as active) on Rev helical hairpin (resi. 9-65) and concave inner surface of Impβ ^(2)^ |

| **Distance restraints** |  |  |  |  |
| --- | --- | --- | --- | --- |
|  | **Experiment 1** | **Experiment 2** | **Experiment 3** | **Experiment 4** |
| Compensatory Mutagenesis | D288^Impβ^(Cγ) : R42^Rev^(Cζ) ≤ 5 Å | Same as Expt. 1 | D288^Impβ^(Cγ) : R42^Rev^(Cζ) ≤ 5 Å | Same as Expt. 3 |
|  | D288^Impβ^(Cγ) : R46^Rev^(Cζ) ≤ 5 Å |  | D288^Impβ^(Cγ) : R46^Rev^(Cζ) ≤ 5 Å |  |
|  |  |  | E437^Impβ^(Cδ) : R48^Rev^(Cζ) ≤ 6 Å |  |
| BS3 Crosslinking | K23(Cβ)^Impβ^ : K20(Cβ)^Rev^ ≤ 30 Å | K854(Cβ)^Impβ^ : K20(Cβ)^Rev^ ≤ 30 Å | Same as Expt. 1 | Same as Expt. 2 |
|  | K62(Cβ)^Impβ^ : K20(Cβ)^Rev^ ≤ 30 Å | K857(Cβ)^Impβ^ : K20(Cβ)^Rev^ ≤ 30 Å |  |  |
|  | K68(Cβ)^Impβ^ : K20(Cβ)^Rev^ ≤ 30 Å | K85*9*(Cβ)^Impβ^ : K20(Cβ)^Rev^ ≤ 30 Å |  |  |
|  |  | K867(Cβ)^Impβ^ : K20(Cβ)^Rev^ ≤ 30 Å |  |  |
|  |  | K873(Cβ)^Impβ^ : K20(Cβ)^Rev^ ≤ 30 Å |  |  |

**B. Docking results**

|  | **Experiment 1** | **Experiment 2** | **Experiment 3** | **Experiment 4** |
| --- | --- | --- | --- | --- |
| Total structures clustered: ^(3)^ | 143 | 177 | 84 | 189 |
| No. clusters: | 9 | 11 | 7 | 7 |

**Docking Statistics ^(4)^:**

|  | **Rank** | **Cluster ID**  **^(5)^** | **Cluster size** | **Haddock Score**  **(kcal/mol)** | **Z-score** | **rmsd from**  **LES (Å) ^(6)^** | **Van der Waals**  **Energy**  **(kcal/mol)** | **Electrostatic**  **Energy**  **(kcal/mol)** | **Desolvation**  **Energy**  **(kcal/mol)** | **Restraints Violation**  **Energy**  **(kcal/mol)** | | **Buried**  **Surface**  **Area (Å^2^)** |
| --- | --- | --- | --- | --- | --- | --- | --- | --- | --- | --- | --- | --- |
| **Expt.1** | 1 | 1 | 61 | -160.2 ± 3.6 | -1.5 | 1.2 ± 0.2 | -37.9 ± 1.5 | -775.9 ± 71.5 | 32.8 ± 15.8 | 0.3 ± 0.3 | | 1864.9 ± 110.6 |
|  | 2 | 3 | 15 | -149.7 ± 14.2 | -1.1 | 0.9 ± 0.5 | -42.7 ± 7.0 | -723.4 ± 63.5 | 37.7 ± 7.5 | 0.0 ± 0.1 | | 1876.8 ± 176.1 |
|  | 3 | 4 | 9 | -145.5 ± 15.0 | -1.0 | 1.6 ± 0.3 | -37.1 ± 7.8 | -672.8 ± 67.8 | 26.2 ± 8.1 | 0.0 ± 0.0 | | 2121.8 ± 173.4 |
|  | 4 | 9 | 4 | -123.8 ± 27.7 | 0.1 | 2.1 ± 0.1 | -33.4 ± 10.7 | -642.4 ± 102.0 | 38.1 ± 8.6 | 0.0 ± 0.0 | | 2058.5 ± 268.9 |
|  | 5 | 2 | 34 | -122.9 ± 16.0 | 0.1 | 2.0 ± 0.4 | -26.1 ± 9.2 | -516.9 ± 72.5 | 6.6 ± 6.5 | 0.0 ± 0.0 | | 1360.0 ± 259.5 |
|  | 6 | 5 | 7 | -106.4 ± 8.9 | 0.6 | 8.0 ± 0.1 | -35.6 ± 9.5 | -425.7 ± 63.9 | 11.4 ± 8.3 | 29.0 ± 17.0 | | 1728.4 ± 56.0 |
|  | 7 | 8 | 4 | -100.8 ± 13.5 | 0.8 | 2.0 ± 0.2 | -32.5 ± 3.6 | -428.5 ± 11.1 | 17.4 ± 10.5 | 0.1 ± 0.1 | | 1620.8 ± 169.2 |
|  | 8 | 6 | 5 | -99.3 ± 12.2 | 0.8 | 1.8 ± 0.2 | -25.9 ± 9.8 | -485.1 ± 55.2 | 23.6 ± 3.0 | 0.0 ± 0.0 | | 1674.0 ± 247.3 |
|  | 9 | 7 | 4 | -77.5 ± 16.1 | 1.7 | 1.7 ± 0.2 | -13.8 ± 3.4 | -395.2 ± 53.7 | 15.3 ± 9.5 | 0.0 ± 0.0 | | 1018.2 ± 102.2 |
| **Expt.2** | 1 | 1 | 68 | -169.1 ± 11.7 | -2.3 | 0.7 ± 0.4 | -48.7 ± 5.7 | -584.8 ± 102.0 | 3.5 ±  8.2 | 0.1 ± 0.1 | 2109.8 ± 115.6 | |
|  | 2 | 8 | 6 | -139.2 ± 18.1 | -0.8 | 3.4 ± 0.1 | -42.3 ±  5.4 | -643.4 ± 45.4 | 28.8 ± 11.5 | 29.2 ± 16.0 | 2042.9 ± 95.2 | |
|  | 3 | 2 | 48 | -136.0 ± 1.9 | -0.6 | 1.9 ± 0.2 | -39.9 ± 8.2 | -494.2 ± 53.1 | 2.7 ±  8.9 | 0.0 ± 0.0 | 1718.7 ± 69.1 | |
|  | 4 | 3 | 11 | -126.2 ± 3.3 | -0.1 | 2.4 ± 0.1 | -38.5 ± 5.0 | -477.1 ±  94.4 | -0.7 ± 19.1 | 83.5 ± 21.1 | 1821.9 ± 154.8 | |
|  | 5 | 6 | 7 | -124.0 ± 2.7 | 0.0 | 3.2 ± 0.1 | -27.1 ± 6.2 | -552.3 ± 51.5 | 12.8 ±  8.1 | 7.8 ± 12.6 | 1518.3 ± 101.6 | |
|  | 6 | 4 | 9 | -116.6 ± 7.4 | 0.4 | 2.6 ± 0.4 | -34.6 ± 5.3 | -469.8 ± 61.5 | 11.9 ± 16.6 | 0.0 ± 0.0 | 1572.2 ± 172.6 | |
|  | 7 | 7 | 6 | -111.7 ± 10.6 | 0.6 | 4.8 ± 0.1 | -43.3 ± 8.4 | -511.8 ± 26.6 | 11.9 ± 16.6 | 173.2 ± 6.3 | 1951.9 ± 63.9 | |
|  | 8 | 5 | 9 | -107.0 ± 18.5 | 0.9 | 1.7 ± 0.1 | -24.6 ±  4.2 | -346.4 ± 95.5 | -13.1 ± 6.8 | 0.5 ± 0.5 | 1198.4 ± 73.8 | |
|  | 9 | 9 | 5 | -105.2 ± 16.5 | 1.0 | 2.1 ± 0.3 | -32.1 ± 10.9 | -368.7 ± 77.5 | -2.0 ± 10.9 | 26.4 ± 32.9 | 1548.7 ± 191.1 | |
|  | 10 | 10 | 4 | -102.2 ± 30.6 | 1.1 | 2.9 ± 0.1 | -45.4 ±  4.8 | -356.8 ± 74.3 | 13.4 ± 15.2 | 11.8 ± 19.9 | 1692.1 ± 171.5 | |
|  | 11 | 11 | 4 | -98.0 ± 23.5 | 1.3 | 2.0 ± 0.1 | -44.2 ± 10.2 | -395.8 ± 89.1 | 25.3 ± 9.6 | 0.0 ± 0.1 | 1696.3 ± 250.8 | |
| **Expt. 3** | 1 | 1 | 33 | -142.4 ± 15.6 | -1.7 | 0.7 ± 0.4 | -62.1 ± 8.8 | -627.7 ± 90.7 | 8.9 ± 13.9 | 363.5 ± 28.3 | | 2479.9 ± 117.0 |
|  | 2 | 2 | 17 | -113.7 ± 4.7 | -0.8 | 8.7 ± 0.2 | -34.1 ± 4.7 | -582.0 ± 39.8 | 21.8 ± 5.7 | 150.2 ± 3.0 | | 1932.6 ± 59.9 |
|  | 3 | 3 | 13 | -109.2 ± 18.8 | -0.6 | 1.3 ± 0.1 | -42.6 ± 3.9 | -622.1 ± 123.8 | 23.1 ± 11.2 | 347.2 ± 51.2 | | 2284.3 ± 97.8 |
|  | 4 | 5 | 5 | -77.6 ± 12.6 | 0.5 | 6.9 ± 0.0 | -34.6 ± 8.4 | -482.4 ± 68.8 | 24.5 ± 24.1 | 290.1 ± 16.1 | | 1783.7 ± 179.7 |
|  | 5 | 4 | 8 | -73.5 ± 11.0 | 0.6 | 9.8 ± 0.2 | -35.0 ± 5.8 | -543.6 ± 42.1 | 16.6 ±  8.2 | 536.8 ± 40.2 | | 1568.3 ± 114.1 |
|  | 6 | 6 | 4 | -71.4 ± 16.5 | 0.7 | 2.9 ± 0.1 | -47.2 ± 5.7 | -526.5 ± 28.7 | 56.7 ± 8.6 | 243.6 ± 20.5 | | 2312.0 ± 84.6 |
|  | 7 | 7 | 4 | -49.2 ± 14.7 | 1.4 | 2.6 ± 0.2 | -23.4 ± 10.4 | -523.6 ± 89.9 | 30.1 ± 9.2 | 488.8 ± 20.6 | | 1613.9 ± 198.2 |
| **Expt. 4** | 1 | 1 | 73 | -152.7 ±  9.4 | -1.9 | 2.1 ± 0.1 | -51.1 ± 3.1 | -546.2 ± 61.3 | -0.7 ± 16.2 | 82.9 ± 18.1 | | 2037.3 ± 76.3 |
|  | 2 | 5 | 13 | -135.5 ± 8.7 | -0.3 | 1.7 ± 0.1 | -29.9 ± 1.8 | -652.4 ± 66.4 | 13.7 ± 6.6 | 112.1 ± 16.2 | | 1750.2 ± 162.6 |
|  | 3 | 2 | 43 | -134.2 ± 7.6 | -0.2 | 1.6 ± 0.3 | -43.3 ± 1.8 | -518.9 ± 52.7 | 11.8 ± 10.5 | 10.7 ± 16.0 | | 1776.9 ± 102.0 |
|  | 4 | 3 | 27 | -133.8 ± 6.6 | -0.1 | 2.2 ± 0.1 | -34.2 ± 1.6 | -642.2 ± 78.0 | 226.1 ± 13.8 | 27.1 ± 14.7 | | 2004.5 ± 97.4 |
|  | 5 | 6 | 10 | -132.3 ± 23.8 | 0.0 | 0.9 ± 0.6 | -37.9 ± 1.2 | -549.2 ± 82.5 | 15.4 ± 15.7 | 1.6 ±  2.0 | | 1701.2 ± 102.4 |
|  | 6 | 7 | 4 | -121.4 ± 16.0 | 1.1 | 1.5 ± 0.1 | -32.7 ± 8.6 | -559.3 ± 88.4 | 19.7 ± 12.7 | 34.1 ± 39.4 | | 1710.7 ± 212.3 |
|  | 7 | 4 | 19 | -118.0 ± 4.2 | 1.4 | 1.8 ± 0.2 | -40.5 ± 4.3 | -494.2 ± 55.3 | 19.4 ± 12.3 | 18.8 ± 31.0 | | 1739.5 ± 122.0 |
|  |  |  |  |  |  |  |  |  |  |  | |  |

^1^ Active residues are those designated as being explicitly located in the intermolecular interface, while passive residues are those designated as potentially but not necessarily in the interface.

^2^ These consist of Impβ residues 8-19, 21-23, 25- 27, 29-31, 33-37, 50-52, 55-56, 59-60, 62-64, 66-79, 81-82, 102-105, 107-108, 110-111, 114-115, 118-119, 122-125, 140-145, 147-149, 152, 155-156, 158-165, 167-169, 184, 186-189, 192-193, 195-197, 199-200, 203-204, 206-207, 209- 212, 229-232, 235-236, 239, 242-243, 246-251, 253-254, 271-275, 277-278, 280-281, 284-285, 288-293, 295-299, 301-313, 315-317, 328-344, 346-347, 350-351, 353-354, 357-362, 377-385, 388, 392, 395-400, 402-403, 420-423, 425-427, 429-430, 434, 436-441, 444, 460, 463-466, 468-469, 471-472, 475-477, 479-484, 486-487, 489-494, 517-518, 520-523, 525-527, 530-531, 533-534, 537-538, 541, 544, 565-572, 574-576, 578-579, 581-583, 585-586, 589-590, 593-598, 613, 616-627, 630, 633-634, 636-642, 644, 660-666, 668-669, 671-673, 675-676, 678-684, 702-709, 711-712, 715-716, 718-719, 722-727, 748-757, 759-760, 763-764, 767-768, 770-771, 774-784, 786, 805-806, 808-813, 816-817, 819-821, 824, 827-828, 829-831, 834-835, 837-839, 841-842, 844-845, 848-849, 851-857, 859-861, 863-864, 866-876.

^3^ Number of structures clustered (out of 200) in the final refinement stage of docking. Structures are clustered if they have a pairwise RMSD < 7.5 Å.

^4^ Docking statistics reported (HADDOCK score, rmsd, energy terms and buried surface area) represent the mean value and standard deviation for the four lowest energy structures in each cluster.

^5^ Cluster ID is the rank of the cluster when ranked according to cluster size.

^6^ LES: lowest-energy structure.
