# Supplementary material for: Binding stoichiometry and structural model of the HIV-1 Rev/importin β complex
Source: Life Sci Alliance. 2022 Aug 22;5(10):e202201431. doi: 10.26508/lsa.202201431 (PMC9396022; doi:10.26508/lsa.202201431)
Supplement: Supplementary file 4 [file LSA-2022-01431_TableS4.docx]

**Table S4. Statistics for rigid body docking of Rev at the C-site.**

| **A. Cβ-Cβ Distance constraints** |  |  |
| --- | --- | --- |
|  | **Compensatory Mutagenesis** | **BS3 Crosslinking** |
|  | D288^Impβ^ : R42^Rev^ ≤ 15 Å | K537^Impβ^ : K20^Rev^ ≤ 30 Å |
|  | D288^Impβ^ : R43^Rev^ ≤ 15 Å | K854^Impβ^ : K20^Rev^ ≤ 30 Å |
|  | D288^Impβ^ : R46^Rev^ ≤ 15 Å | K857^Impβ^ : K20^Rev^ ≤ 30 Å |
|  | E289^Impβ^ : R46^Rev^ ≤ 15 Å | K859^Impβ^ : K20^Rev^ ≤ 30 Å |
|  | E299^Impβ^ : R42^Rev^ ≤ 15 Å | K867^Impβ^ : K20^Rev^ ≤ 30 Å |
|  | E299^Impβ^ : R43^Rev^ ≤ 15 Å | K873^Impβ^ : K20^Rev^ ≤ 30 Å |
|  | E299^Impβ^ : R46^Rev^ ≤ 15 Å |  |
|  | E437^Impβ^ : R48^Rev^ ≤ 15 Å |  |

| **B. Docking Results** | | |  | | | | |  | | | | | | | |
| --- | --- | --- | --- | --- | --- | --- | --- | --- | --- | --- | --- | --- | --- | --- | --- |
|  | **Minimal distance between Impβ and Rev side chains (Å) ^(1)^** | | | | | | | | | | **mean sc/sc** | **Change in Rev vs. Rank1^(3)^** | | |  |
| **Rank** | **D288^Impβ^**  **: R42^Rev^** | **D288^Impβ^**  **: R43^Rev^** | | **D288^Impβ^**  **: R46^Rev^** | **E289^Impβ^**  **: R46^Rev^** | **E299^Impβ^**  **: R42^Rev^** | **E299^Impβ^**  **: R43^Rev^** | | **E299^Impβ^**  **: R46^Rev^** | **E437^Impβ^**  **: R48^Rev^** | **distance^(2)^**  **(Å)** | **Angle**  **(^o^)** | **Shift^(4)^**  **(Å)** |  |  |
| 1 | 2.96 | 2.30 | | 3.55 | 8.01 | 5.61 | 12.77 | | 5.47 | 3.24 | 4.61 | - | - |  |  |
| 2 | 2.30 | 2.30 | | 2.72 | 6.11 | 7.14 | 12.93 | | 4.72 | 8.83 | 4.66 | 60.7 | 2.9 |  |  |
| 3 | 2.69 | 2.30 | | 2.30 | 7.23 | 9.13 | 14.61 | | 7.42 | 3.98 | 4.87 | 15.0 | 4.4 |  |  |
| 4 | 3.77 | 2.30 | | 5.13 | 7.57 | 3.48 | 13.44 | | 2.83 | 7.06 | 5.10 | 33.0 | 3.5 |  |  |
| 5 | 4.71 | 2.30 | | 5.17 | 9.56 | 6.43 | 8.56 | | 3.55 | 4.16 | 5.15 | 33.0 | 1.9 |  |  |
| 6 | 4.79 | 3.37 | | 2.55 | 7.86 | 8.00 | 14.27 | | 6.05 | 3.52 | 5.37 | 29.0 | 4.7 |  |  |
| 7 | 5.03 | 2.30 | | 6.68 | 9.63 | 2.30 | 11.12 | | 2.39 | 4.94 | 5.41 | 0.0 | 5.7 |  |  |
| 8 | 4.54 | 2.60 | | 6.33 | 10.06 | 2.64 | 12.14 | | 3.11 | 9.06 | 5.80 | 29.0 | 5.0 |  |  |
| 9 | 7.47 | 2.30 | | 6.81 | 8.79 | 2.30 | 8.32 | | 2.30 | 6.98 | 5.97 | 33.0 | 5.3 |  |  |
| 10 | 7.84 | 2.30 | | 6.00 | 7.98 | 5.28 | 7.24 | | 2.30 | 7.19 | 6.00 | 29.0 | 3.0 |  |  |
| 11 | 3.72 | 3.93 | | 4.80 | 9.86 | 9.34 | 13.61 | | 7.37 | 5.35 | 6.10 | 30.0 | 7.3 |  |  |
| 12 | 5.47 | 2.30 | | 6.61 | 10.66 | 9.41 | 9.06 | | 4.09 | 7.44 | 6.34 | 66.8 | 6.4 |  |  |
| 13 | 5.47 | 3.71 | | 6.57 | 11.34 | 7.52 | 11.05 | | 4.58 | 6.00 | 6.48 | 44.0 | 6.6 |  |  |
| 14 | 6.70 | 4.51 | | 6.54 | 10.75 | 2.67 | 12.82 | | 3.01 | 8.84 | 6.72 | 32.1 | 3.7 |  |  |
| 15 | 8.92 | 2.30 | | 5.81 | 8.93 | 11.55 | 9.84 | | 5.66 | 6.36 | 7.24 | 33.0 | 7.4 |  |  |
|  |  |  | |  |  |  |  | |  |  |  |  |  |  |  |
|  |  |  | |  |  |  |  | |  |  | **Mean:** | **33.4 ± 15.8** | **4.8 ± 1.7** |  |  |

^1^ Rotamer combinations yielding a side chain separation less than 2.3 Å were assigned a minimal distance of 2.3 Å.

^2^ ΔΔ*p*IC_50_-weighted mean distance between side chains for the 8 pairs of Impβ and Rev residues, calculated as Σ(ΔΔ*p*IC_50,i_*D_i_)/Σ(ΔΔ*p*IC_50,i_), where D_i_ is the minimal distance for the i^th^ pair of Impβ and Rev side chains and ΔΔ*p*IC_50,I_ is the corresponding ΔΔ*p*IC_50_ value.

^3^ The change in the orientation and position of Rev compared to those in the top-ranked structure.

^4^ Distance between the centroids of the two Rev monomers compared.
