# Supplementary material for: Binding stoichiometry and structural model of the HIV-1 Rev/importin β complex
Source: Life Sci Alliance. 2022 Aug 22;5(10):e202201431. doi: 10.26508/lsa.202201431 (PMC9396022; doi:10.26508/lsa.202201431)
Supplement: Supplementary file 5 [file LSA-2022-01431_TableS5.docx]

**Table S5. Summary of final HADDOCK docking experiment with Rev at the C-site.**

**A. Docking parameters**

| **General parameters** |  |
| --- | --- |
| PDB entries used | 2X7L (Rev), 1UKL (Impβ) |
| Active residues^(1)^ | Rev residues R42, R43, R46, R48; Impβ residues D288, D289, D299, E437 |
| Passive residues^(1)^ | All solvent-accessible residues (except those designated as active) on Rev helical hairpin (resi. 9-65) and concave inner surface of Impβ ^(2)^ |

| **Distance restraints** |  |  |  |  |
| --- | --- | --- | --- | --- |
|  | **Compensatory Mutagenesis** | **BS3 Crosslinking** |  |  |
|  | D288^Impβ^(Cγ) : R42^Rev^(Cζ) ≤ 5 Å | K537(Cβ)^Impβ^ : K20(Cβ)^Rev^ ≤ 30 Å |  |  |
|  | D288^Impβ^(Cγ) : R43^Rev^(Cζ) ≤ 5 Å | K854(Cβ)^Impβ^ : K20(Cβ)^Rev^ ≤ 30 Å |  |  |
|  | D288^Impβ^(Cγ) : R46^Rev^(Cζ) ≤ 5 Å | K857(Cβ)^Impβ^ : K20(Cβ)^Rev^ ≤ 30 Å |  |  |
|  | E289^Impβ^(Cδ) : R46^Rev^(Cζ) ≤ 8 Å | K85*9*(Cβ)^Impβ^ : K20(Cβ)^Rev^ ≤ 30 Å |  |  |
|  | E299^Impβ^(Cδ) : R42^Rev^(Cζ) ≤ 8 Å | K867(Cβ)^Impβ^ : K20(Cβ)^Rev^ ≤ 30 Å |  |  |
|  | E299^Impβ^(Cδ) : R43^Rev^(Cζ) ≤ 8 Å | K873(Cβ)^Impβ^ : K20(Cβ)^Rev^ ≤ 30 Å |  |  |
|  | E299^Impβ^(Cδ) : R46^Rev^(Cζ) ≤ 6 Å |  |  |  |
|  | E437^Impβ^(Cδ) : R48^Rev^(Cζ) ≤ 6 Å |  |  |  |

**B. Docking results ^(3)^**

| **Rank** | **Cluster** | **Cluster** | **Haddock** | **Z-** | **rmsd vs.** | **Van der** | **Electrostatic** | **Desolvation** | **Restraints** | **Buried** | **Change in Rev vs. LES_1_ ^(7)^** | |
| --- | --- | --- | --- | --- | --- | --- | --- | --- | --- | --- | --- | --- |
|  | **ID**  **^(4)^** | **Size**  **^(5)^** | **Score**  **(kcal/mol)** | **score** | **LES ^(6)^**  **(Å)** | **Waals**  **Energy**  **(kcal/mol)** | **Energy**  **(kcal/mol)** | **Energy**  **(kcal/mol)** | **Violation**  **Energy**  **(kcal/mol)** | **Surface**  **Area (Å^2^)** | **Angle**  **(^o^)** | **Shift^(8)^**  **(Å)** |
| 1 | 1 | 112 | -166.7 ±  4.6 | -1.9 | 0.7 ± 0.4 | -36.8 ± 3.7 | -781.9 ± 50.9 | 10.2 ± 6.3 | 162.9 ± 39.6 | 1927.1 ± 76.7 | 7.2 ± 2.5 | 1.5 ± 0.5 |
| 2 | 5 | 9 | -146.2 ± 11.8 | -1.0 | 1.3 ± 0.1 | -41.2 ± 5.1 | -825.8 ± 41.2 | 40.7 ± 15.7 | 194.1 ± 17.8 | 2326.1 ± 64.0 | 46.5 ± 7.5 | 1.8 ± 0.8 |
| 3 | 3 | 19 | -123.7 ± 6.9 | 0.0 | 1.3 ± 0.3 | -29.1 ± 9.5 | -681.8 ± 88.2 | 20.3 ± 21.2 | 214.6 ± 57.5 | 1610.3 ± 103.0 | 24.2 ± 5.2 | 3.4 ± 1.7 |
| 4 | 2 | 27 | -116.7 ± 4.5 | 0.3 | 1.7 ± 0.4 | -31.1 ± 6.5 | -624.9 ± 88.9 | 23.0 ± 12.7 | 164.2 ± 27.5 | 1635.2 ± 138.9 | 58.2 ± 5.3 | 4.6 ± 0.5 |
| 5 | 4 | 16 | -112.2 ± 4.4 | 0.5 | 2.0 ± 0.2 | -20.6 ± 3.5 | -632.1 ± 52.1 | 17.4 ± 15.3 | 174.9 ± 52.3 | 1484.8 ± 163.1 | 34.9 ± 0.9 | 3.0 ± 0.3 |
| 6 | 6 | 6 | -106.9 ± 7.2 | 0.7 | 2.1 ± 0.4 | -46.3 ± 7.1 | -519.6 ± 50.4 | 17.1 ± 18.3 | 261.9 ± 27.9 | 1726.7 ± 148.6 | 24.3 ± 9.3 | 5.8 ± 1.0 |
| 7 | 7 | 5 | -94.3 ± 21.2 | 1.3 | 1.9 ± 0.1 | -40.7 ± 6.3 | -541.3 ± 65.2 | 25.6 ± 10.9 | 290.5 ± 45.1 | 2068.5 ± 97.6 | 16.9 ± 1.4 | 5.6 ± 0.2 |
|  | **Total:** | **194** |  |  |  |  |  |  |  | **Mean:** | **34.2 ± 16.2** | **4.0 ± 1.6** |

^1^ Active residues as those designated as being explicitly located in the intermolecular interface, while passive residues are those designated as potentially but not necessarily in the interface.

^2^ See footnote (2) of Table S3 for explicit list of residues.

^3^ Docking statistics reported (HADDOCK score, rmsd, energy terms and buried surface area) represent the mean value and standard deviation for the four lowest energy structures in each cluster.

^4^ Cluster ID is the rank of the cluster when ranked according to cluster size.

^5^ Number of structures clustered (out of a total of 200 tested) in the final refinement stage of docking. Structures are clustered if they have a pairwise RMSD < 7.5 Å.

^6^ LES: lowest-energy structure.

^7^ The change in the orientation and position of Rev in the four lowest-energy structures of each cluster compared to those in the lowest-energy structure of cluster 1.

^8^ Distance between the centroids of the two Rev monomers compared.
